# Optical Molecular Imaging of Inflammatory Cells in Interventional Medicine–An Emerging Strategy

**DOI:** 10.3389/fonc.2019.00882

**Published:** 2019-09-12

**Authors:** Gavin P. Birch, Thane Campbell, Mark Bradley, Kevin Dhaliwal

**Affiliations:** ^1^EaStChem School of Chemistry, University of Edinburgh, Edinburgh, United Kingdom; ^2^Centre for Inflammation Research, University of Edinburgh, Edinburgh, United Kingdom

**Keywords:** molecular imaging, inflammation, interventional medicine, optical imaging, macrophage, neutrophil

## Abstract

The optical molecular imaging of inflammation is an emerging strategy for interventional medicine and diagnostics. The host's inflammatory response and adaptation to acute and chronic diseases provides unique signatures that have the potential to guide interventions. Thus, there are emerging a suite of molecular imaging and sensing approaches for a variety of targets in this area. This review will focus on two key cellular orchestrators that dominate this area, neutrophils and macrophages, with recent developments in molecular probes and approaches discussed.

## Introduction

Recent developments in optical molecular imaging are enabling the identification and evaluation of inflammatory cells such as macrophages and neutrophils in a variety of imaging scenarios. This is important as these leukocytes are an important part of host defense and immune homeostasis. Their pivotal roles as professional phagocytes in acute and chronic inflammatory disease may allow their use as versatile interventional biomarkers and as such fluorescent imaging of these cells has the potential to advance disease treatment and management, by producing sub-cellular information in real-time.

Intrinsic tissue autofluorescence adds further value when the structures of extracellular matrix or metabolic activities are of interest. The predominant modality of fluorescence intensity measurements, coupled with recent technological developments such as label-free non-linear optical images and Raman analyses (1, 2), have opened up the possibility of giving the surgeon functional images of the surgical site in real time. Furthermore, exciting developments are resulting from the integration of cell classification and machine learning techniques, allowing deeper analysis of the richness of imaging datasets.

Macroscopic widefield open fluorescence systems and high-resolution endomicroscopy are the predominant systems that have been used clinically to detect optical molecular tracers. The recent advances in imaging technology mean that fluorescence guided surgery is becoming a reality for cancer resection (3). Complete tumor removal is crucial for patient outcomes, but difficult to ensure by conventional microscopy as visual characteristics and palpitation are inadequate to determine tumor-free margin. With fluorescence systems, surgeons have the future ability to assess the extent of tumor excision and local metastases in real-time using a fluorescent label that “lights-up” the tumor and hence delineates its margins. Optical endomicroscopy (OEM) enables optical imaging at high resolution typically via a bundle of optical fibers (although rapidly being replaced by chip-on-tip technologies) to enable imaging with microscopic resolution, with the added ability to explore within a variety of cavities. Recently fiber-bundle endomicroscopy has been used in the lung to assess alveolar structure in emphysema, neoplastic changes in epithelial cells and real-time *in vivo* detection of bacteria (4–6). As well as in the GI tract, where it has been used to detect changes associated with squamous cell carcinoma and colorectal polyps (7). There are a range of OEM platforms, spanning clinical use and developmental systems (6, 8), although standardization is necessary to generate meaningful diagnostic data (9).

Alongside the devices that enable real-time fluorescence imaging capability, optical molecular probes are required that provide contrast and are suitable for use *in vivo*. Although extensive libraries of optical agents for many targets have been developed (10, 11), none have been licensed for routine clinical use. This review will focus on fluorescent molecular probes for inflammatory cells, in particular neutrophils and macrophages. Recent agents allow the identification of these cells, as well as providing information on dynamic cellular processes such as enzymatic activity, redox processes, and phagocytic ability; processes that impact pathophysiology. Overall, inflammatory cell imaging creates an ideal paradigm for patient-specific disease monitoring and intervention (Table 1). Recent advances in the fluorescent and Raman imaging are enabling macrophage and neutrophil burden and activity to be described non-invasively and dynamically with tissue-level resolution.

**Table 1:** Evidence for the role of neutrophils and macrophages as biomarkers in various diseases.

**Cell**	**Information**	**Disease**	**References**
Neutrophil	Disease stage	Gastrointestinal tumors	(12)
		Solid tumors	(13)
		Inflammatory bowel disease	(14)
	Disease onset	Bacteremia	(15)
		Sepsis-induced acute kidney injury	(16)
	Critical event	Cardiovascular	(17)
		Diabetic neuropathy	(18)
		Chronic obstructive pulmonary disease	(19)
		Pesticide poisoning	(20)
Macrophage	Disease stage	Solid tumors	(21)
		Sentinel lymph node metastasis (breast cancer)	(22)
		Rheumatoid arthritis	(23)
		Liver fibrosis	(24)

*Data referenced to meta-analyses or patient studies in the literature*.

## Neutrophils as Biomarkers

As cells that migrate to diseased tissues and accumulate, both over the course of a disease and due to chemotactic stimuli, neutrophils can provide meaningful prognostic data just by their enumeration. Neutrophil counts have long been used to stratify patients by scoring disease, for example in ulcerative colitis (25). Several large patient studies and systematic reviews show the neutrophil to lymphocyte ratio is useful in staging inflammatory bowel disease (IBD) and numerous solid tumors (13, 14), as well as predicting cardiovascular disease, diabetic neuropathy, sepsis-induced acute kidney injury, glaucoma and bacteremia (15–19, 26). Simple neutrophil enumeration may even resolve the controversial question of when to intervene in carotid artery disease (27). Using explanted human tissues Ionita et al. evidenced correlations between neutrophil count and several critical factors in atherosclerotic plaque stability (28).

Of course, more sophisticated insights can be drawn from considering the viability status of neutrophils and quantifying neutrophil extracellular traps (NETs) independently of neutrophils in altered activation states. The accumulation of neutrophils on the ocular surface in dry-eye disease (DED) is thought to result in pathological NET formation (29). With the recent success of a phase I/II study evaluating DNase treatment for DED, NET burden is becoming an important clinical parameter in this disease (30). Also, neutrophil nuclei undergo morphological changes in DED that can assist the detection of DED-related hyperosmolarity (31). Due to the abundance of human neutrophil elastase (HNE) in NETs (32) and this enzyme's stimulatory role in mucin production (33), HNE may allow diagnostic disease monitoring in DED. Beyond the conjunctiva, neutrophil infiltration is inversely correlated with neuroprotection D1 and their pro-inflammatory activity has clear implications in retinal vascular and neural degeneration (34). Tumor associated neutrophils (TANs) play a wide array of roles and their investigation can reveal where they act in opposition. For example, in early stage lung cancer TANs were shown to help limit disease progression however animal models have elucidated several pro-tumoral mechanisms (35, 36). Some of the heterogeneity measured may come from relying on the expression of TAN markers which can vary in different tumor microenvironments and no suitable marker exists for distinguishing between the N1 and N2 phenotypes (37).

Another layer of insight may be accessible via measurement of neutrophil activity, as studies of patients with inflammatory bowel disease have demonstrated. Patient studies found changes in fecal lactoferrin, calprotectin and the protease HNE were significantly correlated with endoscopy and could be used to distinguish between mild disease, mucosal healing and clinical remission, and even predict flare onset (38). HNE is further implicated in IBD treatment by cleaving therapeutic monoclonal antibodies Infliximab, Adalumimab, Vedolizumab (39). The varied and clear benefits of such simple and direct analyses highlight the precision of the inflammatory response and the diagnostic potential of optical probes for neutrophil imaging.

## Neutrophil Imaging Probes

Neutrophils are the first leukocytes to be recruited to an inflammatory site. Their capacities to respond en masse and rapidly underscore their potential as biomarkers capable of producing large readouts in short time-frames—properties that are particularly desirable for surgical guidance. The relatively short neutrophil half-life acts to limit any off-target consequences of labeling to days or hours, whilst the persistence of the neutrophilic influx in inflamed tissues makes neutrophil readouts useful over the entire disease monitoring period, irrespective of its duration. Overall, neutrophil imaging creates an ideal paradigm for patient-specific disease monitoring and intervention. Recent advances in the fluorescent and Raman spectroscopic modes of optical imaging are, for the first time, enabling neutrophil burden and activity to be described non-invasively and dynamically with tissue-level resolution (Table 2).

**Table 2:** Overview of optical probes for imaging neutrophils.

**Target**	**Molecule class**	**Optical modality**	**Models**	**References**
Serprocidins	Quenched dendrimeric probe	Fluorescence	Human cells *ex vivo*	(40)
HNE	Quenched probe	Fluorescence	Mouse	(41)
	Quenched dendrimeric probe	Fluorescence	Sheep, human	(42)
FPR1	Peptide	Fluorescence	Human	(43)
	Radioactive nanoparticle	Dual PET/MRI	Mouse	(44)
Nucleus	Label-free	Raman	Human cells *ex vivo*	(45)
	Label-free	2-photon FLIM	Human	(46)

### Proteases

Human neutrophil elastase (HNE) is a serprocidin stored at millimolar concentrations in the azurophil granules of neutrophils (47). Although HNE is chiefly a microbicidal protease, its broad substrate specificity allows neutrophils to use it intracellularly, extracellularly and in membrane-bound form for a variety of purposes. Phagocytosis, extravasation (48), extracellular matrix remodeling (49–51), cell-signaling (52), mucus production (53), mucociliary function, (54), and NETosis (55) all have roles for HNE and a host of further interactions give the enzyme utility for other cell-types including monocytes, endothelial, and adenocarcinoma cells. When released by activated neutrophils HNE can be used to destroy pathogens and promote neovascularization as part of tissue repair, however sustained HNE release contributes to the pathophysiological sequalae of acute respiratory distress syndrome, lung adenocarcinoma, atherosclerosis and other chronic inflammatory diseases.

**Figure 1:**
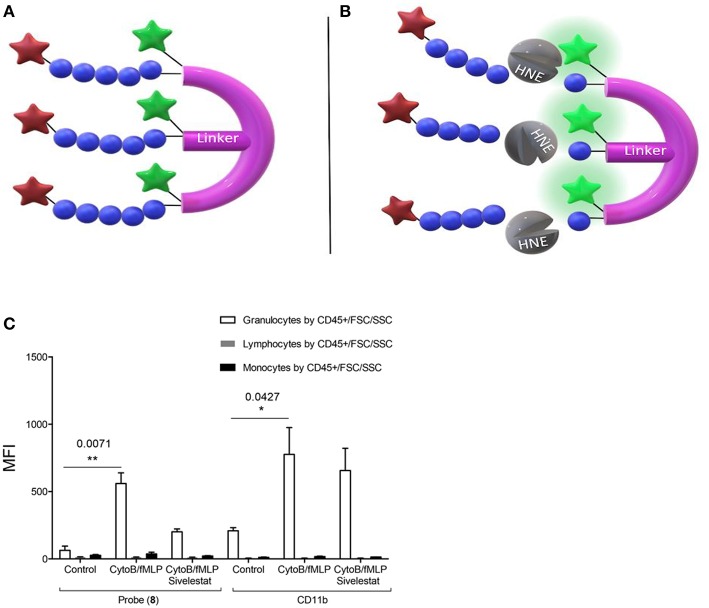
Structure of Craven's neutrophil probe **(A)** before and **(B)** after cleavage by active HNE and indicating the resulting FAM fluorescence. **(C)** In a no-wash, no-lyse whole blood flow cytometric assay, neutrophils activated with bacterial products take up Probe (8) and upregulate CD11b. Fluorescent signal is blocked in activated neutrophils with 100 μM sivelestat co-incubation. MFI = Geometric mean fluorescent index. *P < 0.05, **P < 0.01, exact multiplicity adjusted *p* values are shown with the figure. **(C)** Reproduced under the CC BY 4.0 license (40).

Craven et al. recently reported a probe that revealed neutrophil activation. This was designed to detect the serine proteases (serprocidins) using a pan-serprocidin substrate. A tribranched probe was developed which maintains an optically super-silent ground state with a methyl red and fluorescein FRET pair on each of its three branches (Figure 1A) (40). The structure facilitates internalization by activated neutrophils and once in the phagolysosome, active serprocidins cleave the peptide sequences to remove the methyl red quenchers and generate a large fold increase in fluorescence (Figure 1B). The probe generates bright intracellular puncta, in human neutrophils, within seconds of activation with pharmacological stimulus or bacterial co-incubation. By adding the probe to whole blood in a simple, no-wash, no-lyse, flow cytometric assay, activated neutrophils could be profiled (Figure 1C). Combining rapid signal generation and detailed cell-type specific analysis situates this pan-serprocidin probe as a promising patient-stratification biomarker for several chronic inflammatory diseases.

**Figure 2:**
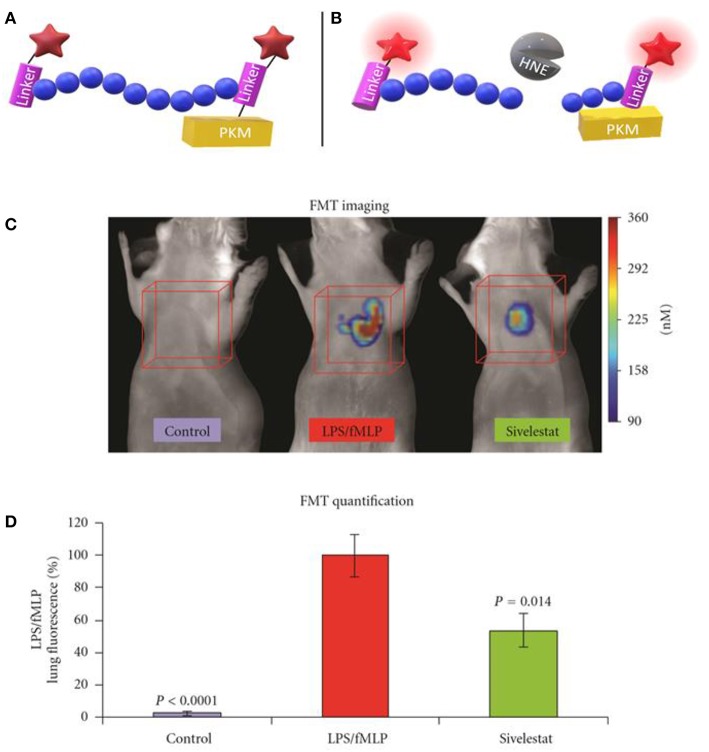
Schematic representation of NE680 FAST before **(A)** and after **(B)** cleavage by active NE which alleviates fluorescence quenching. **(C)** Fluorescence molecular tomography of NE680 FAST (4 nM intranasal) instilled into control, LPS/fMLP and LPS/fMLP mice treated with inhibitor. NIR signal is absent in untreated controls and reduced in sivelestat (5 mg/kg intranasal) treated controls **(D)** and mean concentration of fluorescence (in nM) was quantified in the lung area ROIs (orange cubes) for control (*N* = 12), LPS/fMLP (*N* = 16), and sivelestat (*N* = 12) groups. **(C,D)** Copyright 2011 Kossodo et al. Reproduced under the CC BY 3.0 license (41).

The neutrophil elastase probe NE680 is a near-infrared multi-branched probe which is sensitive to cleavage by murine NE and HNE, amongst others (Figure 2) (41). It consists of a peptide sequence (PMAVVQSVP) flanked by NIR fluorophores and conjugated to a polylysine dendrimer, which lengthens its plasma and tissue half-lives and results in internal quenching. Upon cleavage by proteases, NIR fluorescence emission is generated. NE680's quantification of NE activity was demonstrated by incubating lung sections, from LPS/fMLF challenged mice, in increasing doses of the NE-specific inhibitor, sivelestat (Figures 2C,D). Non-invasive, quantitative NE imaging was demonstrated using fluorescence molecular tomography (41). Wang et al. demonstrated a similar dose dependent reduction in NIR fluorescence of NE680 could be achieved under more physiologically relevant conditions, using recombinant alpha 1-antiproteinase (a1PI) instead of sivelestat (56). Further studies using NE680 have revealed roles for NE in promoting neutrophil accumulation in atherosclerotic plaques, insulin resistance and arthritic pain, in murine models (57–59). Although NE680 cleavage by HNE has been demonstrated *in vitro*, structural differences between the murine and human NE active sites and functional differences between murine and human neutrophils mean the clinical utility of NE680 has yet to be demonstrated.

Although a wealth of neutrophil probes have not reached *in vivo* studies, exciting developments in optical probe design provide discriminatory power between related proteases. Despite their concomitant release from degranulating neutrophils, the various serprocidins perform distinct molecular functions (60). Screening combinations of natural and unnatural amino acids by their kinetic affinity and rate constants, Kasperkiewicz et al. designed a HNE probe with a 100-fold sensitivity over the previous champion substrate designed by Korkmaz et al. (61). The group's combinatorial substrate library technique generated substrate-based activatable probes and inhibitory, targeted probes and their approach included counter selection which biases against the interference of substrate cleavage from similar protease families (62, 63). Finally, the recently synthesized fluorogenic toolbox contained unique substrate-fluorophore combinations for each of the four neutrophil serine proteases (HNE, proteinase 3, cathepsin G and neutrophil serine protease 4) and revealed for the first time their uneven distributions in azurophil granules (64).

To enable clinical, functional neutrophil imaging via HNE activity at inflammatory sites a Neutrophil Activation Probe (NAP) was developed (42). Using static quenching NAP's tribranched structure holds fluorescein moieties in close proximity limiting fluorescence. Each of these SmartProbe's three branches contain an HNE substrate sequence cleaved by the active enzyme to generate large fold increases in fluorescent intensity. Encouraging results with NAP came from synthesizing the SmartProbe to GMP standards and endomicroscopically imaging neutrophil activation in ventilated and perfused *ex vivo* human lungs (37). Craven et al. found NAP to be dequenched within the phagolysosome specifically in response to NE and this lead to a successful phase 1 clinical study (NCT01532024) (42). The ability of NAP to inform clinical decision making is currently being investigated in the phase 2 clinical study, SNAP-IT (number: NCT02804854). SNAP-IT will evaluate the utility of imaging NAP-illumined neutrophils, endomicroscopically, in intensive care unit patients.

### Formyl Peptide Receptor 1

**Figure 3:**
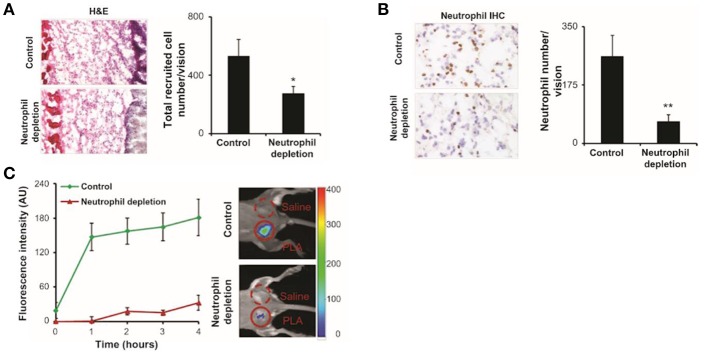
fMLF receptor targeting nanoprobes were implanted in control and neutrophil depleted mice, neutrophils were labeled and quantified. **(A)** Image of hematoxylin and eosin staining and quantification of inflammatory cells. **(B)** Immunohistochemistry and quantification of neutrophils. **(C)** Fluorescence intensities over 4 h post-injection showing the diminished accumulation of the nanoprobe in neutrophil depleted mice compared to control. H&E, hematoxylin and eosin; IHC, immunohistochemistry; PLA, poly(lactic acid); *P < 0.05, **P < 0.01. Copyright 2012 Zhou et al. Reproduced under the CC BY-NC 3.0 license (43).

NIR imaging is often superior to other wavelengths as tissue autofluorescence is lowest in this region of the visible spectrum. Zhou et al. synthesized a NIR fMLF receptor 1 targeting nanoprobe for imaging inflammation (Figure 3) (43). The issue of inflammatory site access was solved by building the labeling (cFLFLF) and fluorophore (Oyster-800) components onto a hydrophilic 8-arm PEG scaffold. There are many benefits to using the cFLFLF ligand: its high affinity FPR1 binding (K_d_ = 2 nM) generates a sensitive readout of leukocyte distribution (65). The ability of cFLFLF probes to access inflammatory sites with either PET (^64^Cu, ^99m^Tc) or NIR (Cy5, Cy7) labels has also been demonstrated. However FPR1 is not cell-type specific and these probes bound macrophages (66) and neutrophils (67, 68). cFLFLF probes may generate a useful readout when information on inflammatory cell accumulation is sought in broad terms but may fail to clarify whether clinical intervention should focus on altering neutrophil or macrophage activity.

Pellico et al. created neutrophil-specific radiotracers with dual PET and MRI signals by coating gallium-doped nanoparticles with cFLFLF for FPR1 labeling (44). Coating with peptide can produce significant probe hydrophobicity which has prevented other radiotracers from reaching the inflammatory site. By citrate-coating nanoparticles, the relatively large nanoparticle surface confers the necessary solubility for targeting neutrophils *in vivo*. Although neutrophil depletion was capable of removing signal, it also removes important cross-talk from the LPS inflammatory response.

A truly neutrophil specific target has proved elusive. Instead neutrophil specificity has arisen from combinatorial strategies exploiting the unique confirmations of certain targets within neutrophils. For example, HNE reaches such high concentrations in neutrophils that most systems will fail to detect the minority of HNE positive monocytes which bares far lower quantities of the enzyme. Neutrophil specific functions can also be exploited by careful probe design. Phagolysosome alkalinisation does not occur in monocytes and enhance the fluorescence signal of some fluorophores. The super-silent FRET probe combines these structural and functional cellular characteristics to achieve neutrophil specificity. Label-free methods of cell identification have also sought neutrophils using targets found in other cell types but uniquely arranged in neutrophils.

### Label Free Neutrophil Imaging

Neutrophil imaging in the context of inflammatory research has aimed at understanding the relevance of cellular components to cell function. Questions of molecular colocalization and characterization are well-served by fluorescent labels for elucidating the spatiotemporal relationships of cells and subcellular structures without altering the processes for investigation. As translational research influences surgical guidance questions of long-term cytotoxicity and ease-of-adoption are more important. Label free imaging, which may seem imprecise for research purposes, is often cheaper, safer and does not require the considerable translation effort of alternative techniques. Molecules such as NADH, elastin, and hemoglobin have been claimed to provide intracellular and extracellular autofluorescence, but signals from cells will always be an amalgamation of signatures of multiple and complex analytes. There is still much characterization to generate clinically meaningful autofluorescence imaging and techniques such as fluorescence lifetime imaging may become an important source of differentiation. Reflectance microscopy, which distinguishes between matter of different refractive indices, is now also capable of cellular resolution. For high-resolution imaging, individual molecules can be detected and quantified via Raman spectroscopy. Label free imaging is en route to becoming as informative as fluorescence image-guided surgery can be, a limit it may exceed in a multi-modal platform.

#### Raman Spectroscopy

By measuring the minute proportion of photons which interact with molecules inelastically, Raman spectroscopy can, non-destructively, describe the chemical composition of unlabeled matter. The first look at a leukocyte using Raman spectroscopy was when Puppels et al. compared eosinophils and Chinese hamster lung cells (69). The group found nuclei spectroscopically distinct from cytoplasm and that distinctions between granulocytes may be made on the basis of their cytoplasmic contents (70). In 1998, Otto et al. collected the first Raman spectrum of activated neutrophils (71). More recently, Ramoji et al. demonstrated the ability to discern between lymphocytes and neutrophils with concomitant Raman mapping of nuclear morphology (45). Using principal components analysis, cells could be classified with 94% accuracy in the validation dataset and predicted with 81% accuracy in a new dataset from a completely different donor. As Raman spectroscopy can describe intracellular contents it may be better suited to quantifying cell function. Instead of identifying cells, Harz et al. used spectrally distinct Raman and fluorescence excitation wavelengths to successfully multiplex these spectroscopic and microscopic techniques (2). Particularly in such a multiplexed system, intracellular localization and oxidation states of functionally significant molecules may be revealed through Raman mapping to imitate whether or not neutrophils are effecting processes such phagocytosis and to what extent (72).

#### Two-Photon Endogenous FLIM

The question of whether or not technology as nascent and complex as two-photon microscopy can be translated into a clinical endomicroscopy technology is unanswered. Although confocal endomicroscopy is in clinical use, two-photon endomicroscopy (TPEM) carries additional fiber optics challenges such as fiber non-linearity distorting the two-photon excitation light and the low quantum yields of intrinsic fluorophores (73). These and other hurdles were overcome in 2008, when TPEM of human and mouse sarcomeres was conducted using a GRIN lens (74). The technique involved a minimally-invasive needle clad fiber capable of imaging directly beneath the skin. *In vivo* TPEM was performed laparoscopically with a “stick” lens which avoids GRIN lens associated spherical aberrations revealing ovarian cancer through a laparoscopic procedure, centimeters into the body (75). However murine tissues were investigated *in vivo* with a flexible FLIM endomicroscope, of working distance 135 μm in 2011, and the field continues to rapidly improve this label-free technology (76).

**Figure 4:**
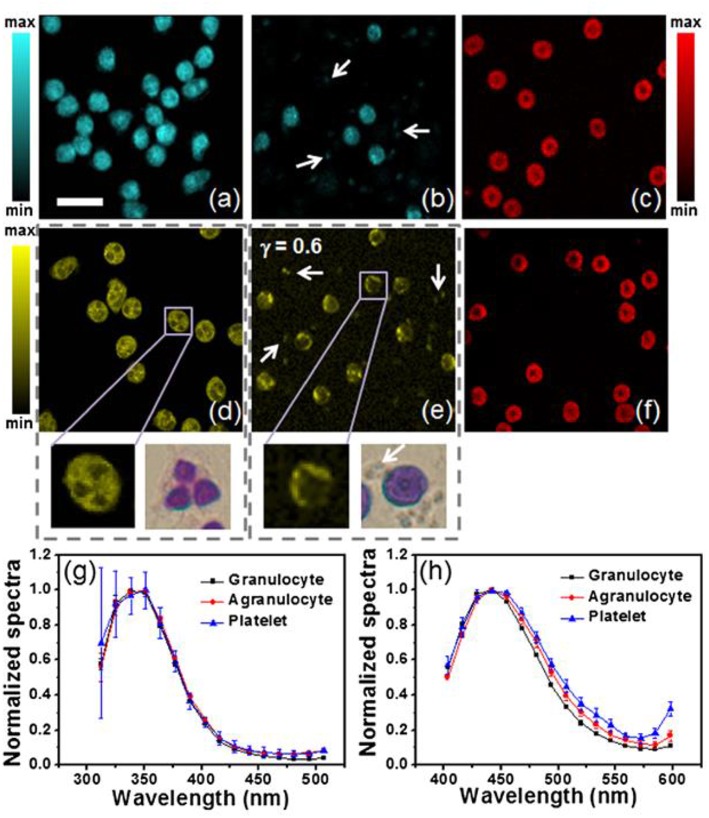
TPEF showing granulocytes, agranulocytes with platelets (white arrows) and erythrocytes **(a–c)** under 600 nM excitation and **(d–f)** under 700 nM excitation, with bright field images in **(d)** and **(e)** showing blood smears granulocytes and agranulocytes with platelets. Normalized spectra of blood cell autofluorescence under 600 nm (5 mW) and 720 nm (10 mW) excitation, are seen in **(g)** and **(h)**, respectively. The acquisition time for each TPEF image: 32 s; resolution: 128 × 128 pixels; scale bar: 20 μm; γ is the gamma value in the gamma correction of **(e)**. Reproduced with permission from Zeng et al. Copyright 2013 SPIE (46).

Using two-photon excited fluorescence (TPEF), Zeng et al. characterized blood cells by their endogenous fluorescence lifetime signals (Figure 4). TPEF fluorescence lifetime imaging can detect differences in bound and unbound NADH such erythrocytes, agranulocytes and granulocytes are distinguishable (46). As proportions of bound and free NADH vary by the dominant metabolic pathway employed by the cell—TPEF FLIM is functional imaging. Metabolic functional imaging may be able to identify granulocytes undergoing phagocytosis but whether or not phagocytosis and other factors affecting the NAD/NADH ratio, such as oxygen availability, can be dis-entangled remains unclear. By visualizing cytoplasmic protein, TPEF FLIM can identify the size and shape of cells and, by exclusion, their nuclei—detailing the characteristic lobular structure of granulocyte nuclei. With neutrophils comprising 95% of the granulocytes population, these morphological distinctions give TPEF FLIM neutrophil-specificity to rival the best ligand-based, fluorescent probes. Although the distinction between phagocytic and untreated neutrophils is subtle, this technique can separate untreated from activated neutrophils on morphological grounds. Quiescent neutrophils have a rounded morphology but membrane ruffling characteristic of neutrophil activation was visualized when neutrophils phagocytosed *E. coli* (46).

## Macrophages as Biomarkers

Macrophages are readily found at the site of inflammation or infection, forming the first defense against pathogens. Although they play a key role in the initiation of defensive inflammation, recent literature suggests they are also responsible for the resolution of inflammation and repair processes (77). Several large studies show the role of macrophages in solid tumors, sentinel lymph node metastasis, rheumatoid arthritis and liver fibrosis (21–24). In ophthalmology, macrophages have been shown to play a role in preventing ocular infection (78) and contribute to corneal wound healing (79) and biomarkers of macrophage activity have been linked to primary open angle glaucoma and dry eye disease (80, 81).

Macrophages have been broadly classified into two different phenotypes—M1 and M2, with further subsets M2a, M2b, M2c identified, and they display plasticity by moving between these phenotypes (82). Generally, the M1 “classically activated” macrophages are seen as pro-inflammatory and exhibit killing mechanisms against microorganisms, while M2 “alternatively activated” macrophages are anti-inflammatory and exhibit wound healing functions. Further evidence shows that they do not rest in one polarization state, instead they may be reactivated into a different state. The ability to understand the crosstalk between macrophage activation signals and how this alters their phenotypes would be valuable in characterizing disease phenotypes. In oncology, tumor associated macrophages (TAMs) represent a significant imaging target due to their recruitment in the microenvironment of a tumor (83). Their presence contributes to the invasiveness of tumors as they have been linked to angiogenesis, cell proliferation, invasion and immune suppression (84). Targets to visualize macrophages include MMP-12, cathepsin S, the macrophage mannose receptor (CD206), and folate receptor beta (FR-β) (Table 3).

**Table 3:** Overview of optical probes for imaging macrophages.

**Target**	**Molecule class**	**Optical modality**	**Models**	**References**
FR-β	Small molecule	SPECT/CT, *in vivo* imaging	Mice/Human (RA)	(85, 86)
CD206	Nanobody (sdAb)	PET	Mice	(87)
	Peptide	Fluorescence	Mice	(88)
MMP-12	Peptidomimetic	Fluorescence	Mice	(89)
Cathepsins	Quenched polymer probe	Fluorescence	Rat	(90)
	Peptide—activity probe	PET/CT and Fluorescence	Mice, Human (PET in IPF patients)	(91)

### Folate Receptor Beta

**Figure 5:**
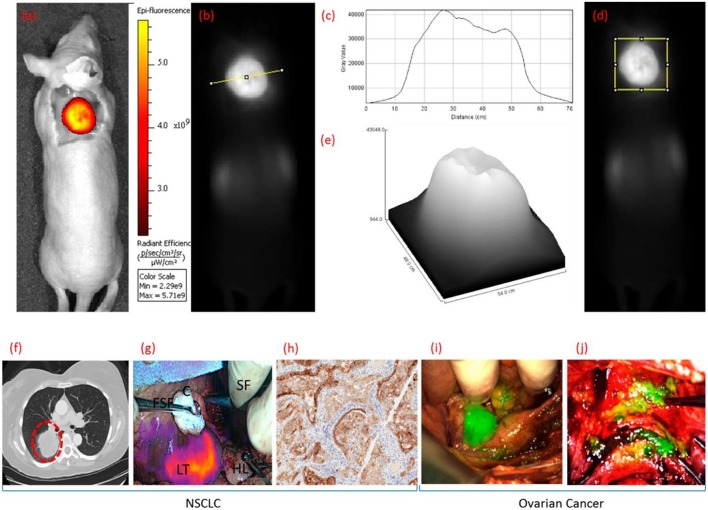
Clinical uses of OTL38 showing whole body fluorescence imaging of a mouse bearing a FRα^+^ KB tumor in color **(a)** and grayscale **(b,d)** 2 h after administering OTL38. Analysis of gray value vs. distance **(c,e)** [across the line from **(b)** or within the box from **(d)**]. Representative fluorescence images NSCLC and ovarian cancer during image-guided surgery 2 h after administering OTL38. **(f)** Preoperative CT image of pulmonary tumor nodal, **(g)** overlay of fluorescence image over white light image of pulmonary tumor nodal, and **(h)** immunohistochemical (IHC) staining of resected pulmonary tumor nodal indicating tumor is FRα+. Representative fluorescence images over white light images of primary and metastatic ovarian tumors in **(i)** uterine adnexa and **(j)** uterus and bladder peritoneum. LT, lung tumor; HL, healthy lung; FSF, Foerster sponge forceps; SF, surgeons fingers; C, cotton. Adapted with permission from Mahalingam et al. Copyright 2018 American Chemical Society (92).

Folate receptor (FR) targeting is a promising method for visualization of cells that overexpress the folate receptor. Recently, a demonstration of the capabilities of folate targeting during fluorescence guided surgery of cancer was described by Mahalingam (92). OTL-38 is an NIR labeled folate that accumulates in cancer tissue, has a high target affinity and enables the visualization of cancer tissue using image-guided surgery (Figure 5). This agent has completed Phase I and II trials for ovarian and lung cancers, and is now in Phase III clinical trials for the detection of FR^+^ ovarian cancer (NCT03180307). For inflammatory cells, the β isoform (FR-β) is expressed on activated macrophages and has a high affinity for its ligand folic acid (K_d_ = 10^−10^ M) (93). Its identification on synovial macrophages in patients with rheumatoid arthritis was first described by Nakashima-Matsushita et al. (94). Studies on both rheumatoid and osteoarthritis in dogs, horses, rats, mice, and humans have demonstrated that essentially all joints experiencing active inflammation uptake folate conjugates (93). A number of studies have reported on the cellular uptake of FR targeted molecules by activated monocytes (95) and macrophages in a number of different diseases (85, 86, 96).

Xia et al. investigated the presence of the folate receptor on activated macrophages from arthritic patients (85). Fluorescence imaging was used to show that the folate receptor was a valid marker for activated macrophages, while non-activated macrophages did not express the folate receptor. After characterizing that bacteria-recruited murine macrophages expressed FR and that these were activated (upregulation of Ly-6C/G, CD80, and CD86), folate-FITC was incubated with the synovial fluid of patients with diagnosed rheumatoid arthritis. A subset of the CD11b^+^ macrophages was found to uptake folate-FITC, and competition experiments showed that this uptake could be inhibited with the non-conjugated folic acid—showing FR specificity. Finally, a technetium-radiolabelled folate (FolateScan: EC20) was utilized to perform SPECT imaging of human inflamed joints in a Phase II trial (NCT00588393).

**Figure 6:**
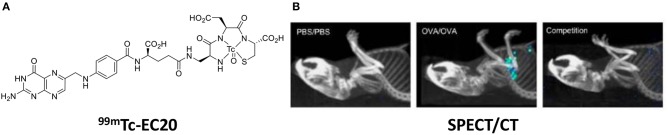
**(A)** Chemical structure of EC20—a folate radiolabel. **(B)** SPECT/CT images ^99m^Tc-EC20 uptake in the lung of OVA-induced asthmatic mice, competition experiment with ^99m^Tc-EC20 plus 100-fold excess folate-glucosamine. Adapted with permission from Shen et al. Copyright 2013 American Chemical Society (86).

In murine models of asthma, Shen et al. showed that *ex vivo* lung macrophages with an M2 phenotype (arginase^+^, CD206^+^) bound the green fluorescent probe, folate-Oregon Green. Further, SPECT/CT imaging of ^99m^Tc-EC20 showed uptake in ovalbumin induced asthmatic lungs, while there was no uptake in the lungs of healthy mice (Figure 6) (86).

Recent work by Poh et al. showed that a folate liposome could act as a specific method of targeting FR^+^ immune cells, specifically in mouse models of colitis and atherosclerosis (97). This study used fluorescent DiD liposomes, labeled with folate, as a demonstration of the ability to deliver payloads via the folate receptor which can accumulate in inflamed tissues. Atherosclerotic mice (ApoE^−/−^ mice fed a high fat diet) were injected via the tail vein with 2 mg/kg of NT-liposome-DiD or Fol-liposome-DiD, respectively. Fluorescence imaging showed selective uptake of Fol-liposomes in the atherosclerotic mice.

To further our understanding of the clinical applicability of folate targeted imaging agents, the field should aim to evaluate their performance in human inflammatory models. It is however also worth mentioning an observation from this field; the use of folate depleted media is necessary for any folate targeting cell studies due to high levels of folic acid in routine media formulations.

### CD206

CD206, also known as the macrophage mannose receptor (MMR), is a well-reported marker of M2 differentiated macrophages (82). Its functional role is to recognize mannose lectins found on pathogens. A number of different approaches to probe design have been taken against CD206^+^ macrophages, from antibodies to a more recent report of a target-binding peptide. The Manocept concept of nanomolecule imaging agents targeting the lectin domain of CD206 has resulted in the FDA approval of γ-Tilmanocept, a ^99m^Tc-labeled radiolabelled tracer for the imaging of sentinel lymph nodes (98). A similar strategy was applied by Kim et al. who developed a near-infrared MMR targeting polymer and utilized using OCT-NIRF imaging to visualize carotid atheroma plaques (99). However, mannose derivatives are not specific to CD206 and can be recognized by other mannose receptors (100). Therefore, there is a need to develop ligands that are more specific for CD206 which are more suitable for use in the clinic.

#### Targeted Nanobodies

The most advanced agents for CD206 targeting are labeled nanobodies, which have shown promise as molecular imaging agents (101). Nanobodies (Nb) have been developed as imaging tracers against a number of targets in oncology and inflammation due to their high affinities and small size compared to antibodies (102). Movahedi et al. produced a ^99m^Tc-labeled nanobody against MMR with an affinity of 2 nM determined by SPR and validated its use for SPECT/micro-CT imaging of tumor-associated macrophages in preclinical models (103). SPECT/CT imaging in tumor-bearing mice showed uptake of the Nb which was significantly higher than uptake of the control Nb with analysis of the dissected tissue confirming these findings.

**Figure 7:**
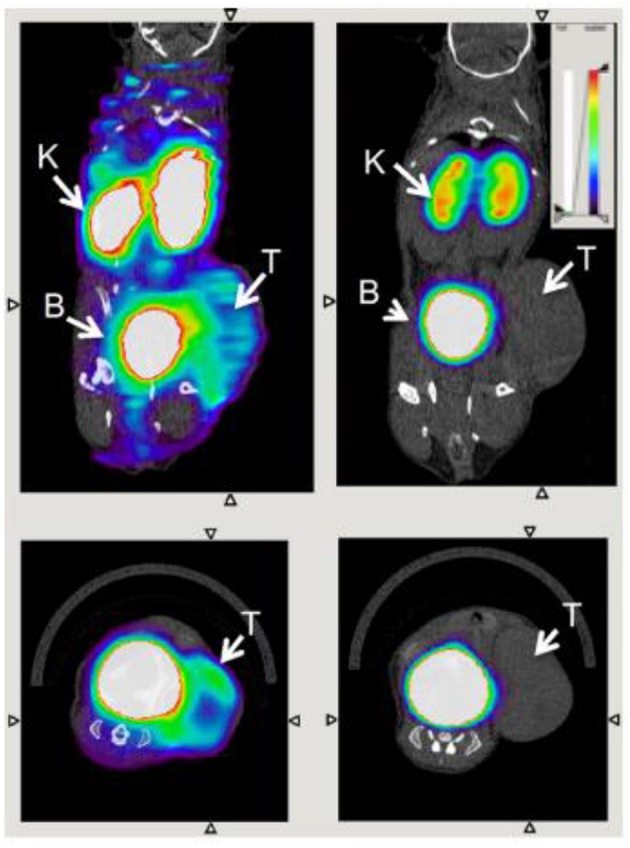
Transverse and coronal PET/CT images of WT (left) vs. MMR-deficient (right) 3LL-R tumor–bearing mice scanned 3 h after injection of ^18^F-FB-anti MMR 3.49. Arrows point to tumor (T), kidney (K), and bladder (B). Adapted with permission from Blykers et al. Copyright 2015 SNMMI (87).

This nanobody was further developed by Blykers et al. as an ^18^F-PET tracer for the detection of macrophages in tumor stroma (87). Tumor associated macrophages (TAMs) with upregulated CD206 were found to be tumor promoting. *In vitro* studies with the ^18^F-fluorobenzoic acid (FB) labeled nanobody (anti-MMR 3.49) showed that it had a high affinity for human MMR (K_D_ = 1.8 nM), while *in vivo* biodistribution studies showed fast renal clearance and specific retention in the tumor and MMR-expressing tissue. In a small animal PET imaging study, the nanobody was specifically recognized by MMR in 3LL-R tumor bearing mice, when compared with the uptake in MMR-deficient mice (Figure 7). This ligand shows promise as a radiopharmaceutical, while in future it could be developed as an optical probe for highlighting M2 macrophages by fluorescence guided surgery. As noted by Debie et al., the fluorophore chosen and method of conjugation should be carefully considered when developing labeled nanobodies (104).

#### Peptides

**Figure 8:**
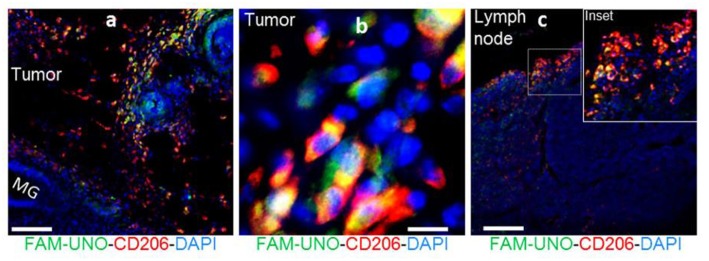
FAM-UNO accumulates in CD206^+^, TIE2^+^ macrophages in breast tumors **(a,b)** and lymph nodes **(c)**. 4T1 tumor bearing mice were injected i.v. with FAM-UNO (30 nmol), mice were sacrificed after 2 h and tumor tissues were analyzed by immunofluorescence: rabbit anti-FAM (green), rat anti-CD206 (red) antibodies and counterstained with DAPI. Scale: 100 μm. Reproduced under the CC BY 4.0 license (88).

Fluorescent peptides are a widely utilized method for labeling receptors and a number of peptidic agents are under clinical investigation for cancer and bacterial infection imaging (10). Recently, Scodeller et al. showed that M2-like TAMs could be targeted by a “FAM-UNO” fluorescent peptide [sequence: 5(6)-FAM-Ahx-CSPGAKVRC] (88). The peptide sequence was identified by *in vivo* phage display of peptides that bound peritoneal macrophages in 4T1 tumor-bearing mice. FAM-UNO was found to target M2-like TAMs via the CD206 receptor (Figure 8) and this binding was confirmed by fluorescence anisotropy. Cargos were delivered to TAMs by coating polymer vesicles with the FAM-UNO peptide and these could be used as a contrast agent for sentinel lymph node imaging. Although this agent is yet to be translated to humans, it could be an attractive method for imaging TAMs. However, green autofluorescence of human tissues means alternative fluorophores may need to be considered to make a case for future clinical applications.

### MMP-12

The matrix metalloproteinases (MMPs) are a family of proteases that play a key role in ECM structure, function and remodeling. In the tumor microenvironment macrophages have been found to be potent producers of MMP 2 and 9 (105). It is important to note the pioneering work of the Tsien lab and Avelas Biosciences in advancing activatable cell penetrating peptides (ACPPs) for imaging protease activity. In 2004, the lab demonstrated that an MMP-2 cleavable ACPP could detect tumor cells in resected tissue (106). More recently, Miampamba et al. developed AVB-620, a ratiometric ACPP with Cy5 and Cy7 fluorophores that is a substrate for MMP-2 and -9 (107). This agent has completed Phase I clinical trials for imaging breast cancer tumors in patients undergoing surgery (NCT02391194).

While there are many non-selective imaging agents for MMPs, there is a lack of selective MMP tracers and this may hamper the understanding of the role of a specific MMP in disease. For example MMP-12, macrophage elastase, is responsible for the breakdown of elastin and it is associated with a number of inflammatory pathologies such as aortic aneurysm, emphysema, and rheumatoid arthritis (108). It has been characterized as having a protective role during wound healing in injury models (109).

**Figure 9:**
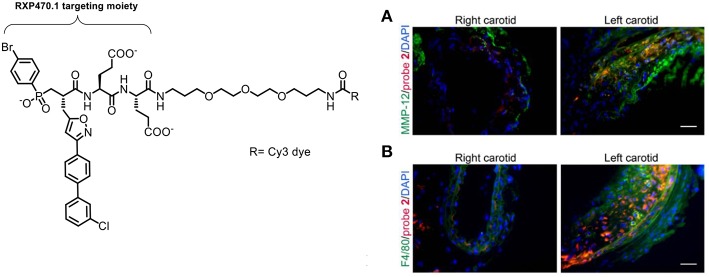
Left: Structure of the Cy3-labeled MMP-12 probe 2. Right: Immunostaining demonstrating that the Cy3-labeled probe 2 (red) binds to carotid arteries and co-localizes with an MMP-12 antibody (**A**, green) and F4/80 positive cells (**B**, green). Nuclei are stained with DAPI (blue). Scale: 50 μm. Reproduced under the CC BY 4.0 license (89).

Bordenave et al. reported the synthesis and evaluation of a Cy5 MMP-12 probe based on a pseudopeptide (RXP470.1) which had previously been shown to inhibit atherosclerotic plaque growth *in vivo* (110). This study noted that the fluorophore used was important to the blood clearance rate and biodistribution of this probe, a factor that has been highlighted by other groups working on the development of fluorescent probes. Further optimization lead to a zwitterionic labeled probe which had good affinity to MMP-12 and fast blood clearance (89). The selectivity of this MMP inhibitor should give advantages over other pan-MMP probes in showing the specific MMP implicated in a disease. Immunostaining showed an analog (probe 2) bound to MMP-12^+^ and F4/80 macrophages (Figure 9). The probe was used for imaging active forms of MMP-12 in murine models. Using a sponge implantation model of sterile inflammation, increased cell infiltration was seen which correlated with mannose receptor positive macrophages. The study was also interested in the role that MMP-12 plays in aneurysm. In a model of carotid aneurysm, significant upregulation of MMP-12 was seen, along with significantly higher signal for the probe, compared to the control (Figure 9). Although this was only demonstrated in a mouse model, because of this probe's high selectivity and the clinical relevance of the experimental setup could allow this probe to be used in an intraoperative setting.

### Cathepsins

TAMs remodel the extracellular matrix (ECM) through MMPs and cathepsins, specifically during tumor invasion (111). Onda et al. used a commercial NIR fluorescent protease-activatable probe (ProSense), in which they demonstrated imaging of cathepsin activity and confirmed its localization to macrophages. Using *in vivo* and *ex vivo* models of colon cancer they showed that infiltrating TAMs initiate tissue remodeling at the tumor margins by secreting cathepsins. ProSense signal at the tumor margin was shown to be due to cathepsin B^+^ macrophage infiltration in a rat colon model (90). However, large probes such as ProSense suffer from slow tumor uptake leading to slow rates of activation, as well as demonstrating off-target activation by other proteases which limits their applicability as a fluorescence diagnostic tool.

A number of alternative cathepsin activatable probes are under development (112, 113). Withana et al. investigated the role that macrophages play in idiopathic pulmonary fibrosis (IPF) by staining human biopsy tissue from IPF patients. The optical probe BMV109 is broadly activated by cathepsins B, S, L, and X, was used to validate pan-cathepsin labeling of sectioned frozen tissue. This showed that macrophages expressing active cathepsins were at fibrotic sites in comparison to healthy tissue which showed no cathepsin activity (91). To date, this probe has only been demonstrated in the topical labeling of IPF tissue, further investigation would be necessary to show its utility in fluorescence based diagnostics in the clinic.

## Conclusion

The nascent field of optical molecular imaging of inflammatory cells appears to be a vibrantly innovative arena full of early-stage biomarkers that offer promise of patient benefits. Targeting neutrophils and macrophages may deliver previously inaccessible measures of disease activity across common and life-threatening diseases. Whilst, in this review, the current stage of neutrophil and macrophage imaging has been discussed, clearly other inflammatory cells such as T cells play major role in interventional medicine. With many non-redundant targets and readouts available, probe multiplexing seems a particular advantage of this field with the potential to matching monitoring and treatment with immunological precision. As well as being informative in its own right, parallel label-free imaging may become a rapid means of interpreting labeled techniques across multiple contexts and machine learning data-reduction stands to push the power of OMI even further. An exciting therapeutic potential lies in the how imaging agents can impact the drug discovery process—with immune cells. These new technologies stand to enrich assay outputs, accelerate drug development decisions and clinical outputs, and enable better direct drug response metrics in trials.

Future studies will demonstrate the translatability of imaging agents into clinically useful optical probes. Despite the plethora of novel reagents advancing with *in vitro* investigations very few have begun the translational journey. This inertia is perhaps not surprising as the technical demands of translating novel imaging methods can become overwhelming, especially within an academic environment. Probes must be innovative enough to meet clinical needs, yet synthetically feasible and complementary to imaging systems. This is a fiercely interdisciplinary pursuit from start to finish and nearly impossible without optical imaging device standards, as all technologies must meet safety and efficacy standards. Without standardization, investigators risk underestimating translation-ready reagents with “sub-standard” apparatus, or unnecessarily pursue doomed reagents with poor selectivity and specificity (114).

## Author Contributions

GB and TC wrote the manuscript. KD and MB assisted with proofreading and preparation for publication.

### Conflict of Interest Statement

MB and KD are shareholders of Edinburgh Molecular Imaging. The remaining authors declare that the research was conducted in the absence of any commercial or financial relationships that could be construed as a potential conflict of interest.

## Abbreviations

DiD: A lipophilic carbocyanine dye
FAM: Carboxyfluorescein
FITC: Fluorescein isothiocyanate
FLIM: Fluorescence lifetime imaging microscopy
FRET: Förster resonant energy transfer
FR: Folate receptor
HNE/NE: (Human) neutrophil elastase
IBD: Inflammatory bowel disease
MMP: Matrix metalloproteinase
MMR: Macrophage mannose receptor
NIR: Near infrared
OEM: Optical endomicroscopy
PET: Positron emission tomography
TPEM: Two-photon endomicroscopy
UC: Ulcerative colitis.
